# Multi-Representation Domain Adaptation Network with Duplex Adversarial Learning for Hot-Rolling Mill Fault Diagnosis

**DOI:** 10.3390/e25010083

**Published:** 2022-12-31

**Authors:** Rongrong Peng, Xingzhong Zhang, Peiming Shi

**Affiliations:** 1 Nonlinear Dynamics and Application Research Center, Nanchang Institute of Science and Technology, Nanchang 330108, China; 2 National Engineering Research Center for Equipment and Technology of Cold Rolled Strip, Yanshan University, Qinhuangdao 066004, China; 3 College of Electrical Engineering, Yanshan University, Qinhuangdao 006004, China

**Keywords:** fault diagnosis, rolling mill, transfer learning, domain adaptation, distribution difference

## Abstract

The multi-process manufacturing of steel rolling products requires the cooperation of complicated and variable rolling conditions. Such conditions pose challenges to the fault diagnosis of the key equipment of the rolling mill. The development of transfer learning has alleviated the problem of fault diagnosis under variable working conditions to a certain extent. However, existing diagnosis methods based on transfer learning only consider the distribution alignment from a single representation, which may only transfer part of the state knowledge and generate fuzzy decision boundaries. Therefore, this paper proposes a multi-representation domain adaptation network with duplex adversarial learning for hot rolling mill fault diagnosis. First, a multi-representation network structure is designed to extract rolling mill equipment status information from multiple perspectives. Then, the domain adversarial strategy is adopted to match the source and target domains of each pair of representations for learning domain-invariant features from multiple representation networks. In addition, the maximum classifier discrepancy adversarial algorithm is adopted to generate target features that are close to the source support, thereby forming a robust decision boundary. Finally, the average value of the predicted probabilities of the two classifiers is used as the final diagnostic result. Extensive experiments are conducted on an experimental platform of a four-high hot rolling mill to collect the fault state data of the reduction gearbox and roll bearing. The experimental results reveal that the method can effectively realize the fault diagnosis of rolling mill equipment under variable working conditions and can achieve average diagnostic rates of up to 99.15% and 99.40% on the data sets of the rolling mill gearbox and bearing, which are respectively 2.19% and 1.93% higher than the rates achieved by the most competitive method.

## 1. Introduction

The rolling mill is indispensable in the production of steel products, and its safe and reliable operation is an effective premise to ensure product quality [1,2]. As modern industrial equipment tends to be large-scale and complex, rolling mill equipment is also developing in the direction of diversified production processes and continuous rolling processes. This complex and variable rolling condition poses great challenges to the condition monitoring and fault diagnosis of rolling mill equipment [3,4]. Under the continuous effect of long-term high load, key components, including a hot rolling mill gearbox, roll bearings, and so on, are prone to failure and damage. If such faults are not detected in a timely manner, they will severely affect the product quality, resulting in considerable economic losses [5].

With the development of artificial intelligence and sensing technology, fault diagnosis has shifted from traditional methods based on expert experience and signal analysis to data-driven fault diagnosis [6]. The support vector machine, random forest, artificial neural network, and other algorithms have made great breakthroughs in solving the traditional problem of relying on complex physical modeling and artificial analysis [7]. However, these fault diagnosis algorithms based on traditional machine learning must be constructed by professionals in feature engineering. The features of these structures are usually only suitable for specific diagnostic tasks and are not universal. In addition, because of the shallow model architecture, traditional machine learning algorithms cannot fully map the nonlinear relationship between state data and fault space.

As a branch of machine learning, deep learning can overcome the lack of nonlinear mapping ability of shallow machine learning algorithms and adaptively learn fault-sensitive features from multiple hidden layers. In recent years, deep learning has been widely reported in the field of fault diagnosis [8,9]. Shao et al. [10] proposed a multi-signal fault diagnosis algorithm based on the convolutional neural network (CNN), which uses vibration and current signals to monitor the state of the motor. For mining the deep-seated state information of mechanical signals, Han et al. [11] used the time- and frequency-domain information together as the model input and proposed an intelligent fault diagnosis method of a dual-stream CNN based on multi-level information fusion. Jia et al. [12] constructed a local connection network through a normalized sparse autoencoder for intelligent fault diagnosis of gearboxes and bearings. Shi et al. [13] studied the health status monitoring of rolling mills based on multi-source sensor fusion under imbalanced and small samples. Yang et al. [14] proposed a residual wide-kernel deep convolutional auto-encoder for intelligent rotating machinery fault diagnosis. Yu et al. [15] developed an approach based on multi-sensor information fusion and improved deep belief networks (DBNs) for the health state diagnosis of rolling mills. The existing literature reveals that the method based on traditional deep learning can achieve superior performance when it can collect sufficient label status data from the target mechanical equipment [16]. However, the actual industrial production process is complex and accompanied by a large amount of environmental noise. The complex and variable working conditions of the hot rolling mill result in the model trained under certain working condition data suffering significant performance degradation when applied for mechanical diagnosis under other working conditions [17].

**Figure 1:**
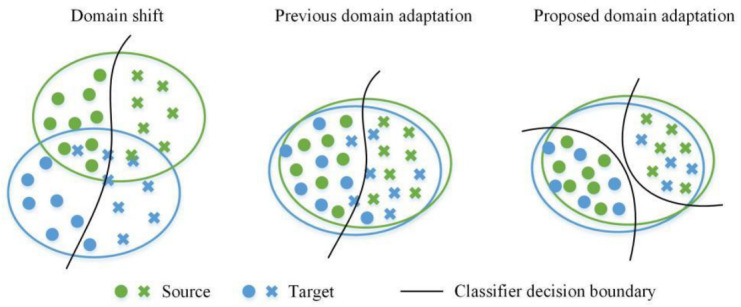
Domain self-adaptive scheme.

The change in data distribution caused by the change in mechanical equipment working conditions is called domain shift [18], as shown in the left panel of Figure 1. Transfer learning is a realistic approach to learning knowledge from one or more tasks and applying it to other related tasks; it can effectively compensate for the differences across domains [19]. In particular, domain adaptation, one of the branches of transfer learning, extracts domain-invariant features through distributed difference measurement or domain adversarial training, which is one of the common algorithms for mechanical condition monitoring and fault diagnosis under variable working conditions [20], as shown in the middle panel of Figure 1. Li et al. [21] used the multi-core maximum mean difference (MMD) to minimize the domain distribution distance in multiple layers of the deep network, which effectively improved the generalization performance of the model. By integrating CORrelation ALignment (CORAL) into a convolutional autoencoder, Qian et al. [22] realized the state recognition of a planetary gearbox under variable working conditions. Li et al. [23] applied the confrontation training method to align the edge distribution and explored the unmarked distribution matching of auxiliary states in parallel data. The bearings at different installation positions were effectively diagnosed. Han et al. [24] proposed a joint distribution domain-adaptive depth transfer network for industrial fault diagnosis, which improved the distribution matching accuracy. Tang et al. [25] added sample label information in the process of domain confrontation and applied conditional distribution domain adaptation to learn domain-invariant features; thus, the accuracy of bearing fault diagnosis was improved. Guo et al. [26] proposed a deep migration learning network with simultaneous MMD measurement and domain confrontation training to maximize the domain recognition error and minimize the probability distribution difference. This process could learn domain-invariant representation. Scholars have applied various domain adaptation methods to mechanical fault diagnosis to promote this research field [27].

Although various domain-adaptive and improved transfer learning methods have alleviated the domain offset problem caused by varying working conditions to a certain extent, the existing domain-adaptive methods only express the transfer diagnosis knowledge from a single piece of information; that is, only part of the mechanical state information is concerned, and the important information related to the machine health may be lost. Thus, the diagnostic performance is unsatisfactory. Literature [28] shows that extracting specific features of observational objects from multiple perspectives can significantly improve the accuracy of cross-domain image classification. To fully transfer health state knowledge from source tasks to target diagnostic tasks, multi-representation information distribution matching should be considered. In addition, owing to the different characteristics of each domain, achieving complete matching of the feature distribution of different domains is difficult, which easily leads to unclear decision boundaries and reduces the accuracy of target diagnosis tasks. To deal with the above two problems, a multi-representation domain adaptation network is proposed in this paper for the diagnosis problem of key equipment of the hot rolling mill under variable conditions. The multi-representation network structure is designed to extract multi-representation information, and the domain adversarial strategy is applied to match the source and target domains represented by each pair simultaneously. This process enables the transfer of sufficient mechanical state knowledge. In addition, the maximum classifier discrepancy is introduced, and adversarial training is introduced to generate target features close to the source support, thereby forming a robust decision boundary, as shown in the right panel of Figure 1. The contributions of this study are as follows:

(1) A multi-representation network structure is designed to fully extract the status information of rolling mill equipment from multiple perspectives.
(2) Domain confrontation and maximum classifier difference discrepancy confrontation training are simultaneously applied to express the transfer of diagnostic knowledge from multiple features and divide the classification boundary of specific tasks.
(3) Extensive experiments are performed to collect the fault state data of the reduction gearbox and roll bearing from a four-high (4-H) hot rolling mill experimental platform. Thus, the effectiveness of the proposed method for rolling mill equipment fault diagnosis under variable working conditions is verified.

The remainder of this paper is organized as follows.

## 2. Preliminaries

### 2.1. Problem Setup

In this study, the general definition of the domain-adaptive fault diagnosis method is followed. Specifically, it is assumed that a tagged source domain dataset $D^{s}={\left\{\left(x_{i}^{s},y_{i}^{s}\right)\right\}}_{i=1}^{n_{s}}$ can be collected under a certain working condition, where $n_{s}$ is the number of source domain samples, and $y_{i}^{s}\epsilon \left\{1,\;2,\;3,\cdots ,\;k\right\}$ represents the corresponding health status tag. The unlabeled data that can be obtained under the working conditions that need to be diagnosed are defined as the target domain $D^{t}={\left\{\left(x_{i}^{t}\right)\right\}}_{i=1}^{n_{t}}$, where $n_{t}$ is the number of samples in the target domain, and $D^{t}$ and$\;D^{s}$ share the same label space. Because of the varying working conditions, such as speed of revolution or load, the distribution of the source domain is inconsistent with that of the target domain, that is, $P\left(X^{s}\right)\neq P\left(X^{t}\right)$. The purpose of fault diagnosis under variable working conditions is to build a cross-domain diagnosis model $y=f\left(x\right)$, which can learn domain-invariance and distinguishability characteristics by eliminating the distribution differences between the two domains and minimize the risk of the target diagnosis task $E_{\left(x,y\right)}\left[f\left(x\right)\neq y\right]$ under source supervision.

### 2.2. Domain Adversarial Training

Domain adversarial training is a typical domain-adaptive method; Ganin et al. [29] first introduced the concept of adversarial training in the field of transfer learning, aiming at minimizing the edge distribution distance of two domains. Specifically, the basic architecture of an adversarial network includes a feature extractor *F* and a domain classifier *D*; usually, a classifier *C* is also included. For a pattern distinguishability problem, their parameters are represented by *θ_F_*, *θ_D_*, and *θ_C_*, respectively. In the training process, feature extractor *F* and domain classifier *D* are two players in a minimax game, that is, domain classifier *D* attempts to identify whether the representation learned by feature extractor *F* originates from the source domain or the target domain, and feature extractor *F* generates cross-domain-invariant characteristics as far as possible to fool domain classifier *D*. In this adversarial training process, the distribution difference between the source domain and the target domain gradually reduces. At the same time, under the supervision of the source domain, classifier *C* is trained to distinguish the categories of different samples. By adding a gradient reverse layer (GRL) to feature extractor *F* and domain classifier *D*, the model optimization of this process can be simultaneously realized.

(1) $$\begin{array}{c} \left({\hat{\theta }}_{F},{\hat{\theta }}_{C}\right)=\arg\underset{\theta_{F},\;\theta_{C}}{\min}l\left(\theta_{F},\;{\hat{\theta }}_{D},\theta_{C}\right) \end{array}$$

(2) $$\begin{array}{c} {\hat{\theta }}_{D}=\arg\underset{\theta_{D}}{\max}l\left({\hat{\theta }}_{F},\theta_{D},{\hat{\theta }}_{C}\right) \end{array}$$

(3) $$\begin{array}{c} l\left(\theta_{F},\;\theta_{D},\theta_{C}\right)=\frac{1}{n_{s}}\underset{x_{i}\in D^{s}}{\sum }J\left(C\left(F\left(x_{i}\right)\right),y_{i}\right)-\frac{\lambda }{n_{s}+n_{t}}\underset{x_{i}\in D^{s}\cap D^{t}}{\sum }J\left(D\left(F\left(x_{i}\right)\right),d_{i}\right) \end{array}$$

where l is the optimization objective of the model, J is the cross-entropy loss function, *y_i_* is the corresponding class label of the source domain sample, *d_i_* is the domain label, and *λ* is the trade-off parameter. ${\hat{\theta }}_{F},{\hat{\theta }}_{D},{\hat{\theta }}_{C}$ are the optimized values of *θ_F_*, *θ_D_*, *θ_C_*, respectively.

### 2.3. Maximum Classifier Discrepancy

Considering that the general domain-adaptive method ignores the relationship between the characteristics of target samples and the task-specific decision boundary, Saito et al. [30] proposed the unsupervised domain-adaptive method of maximum mean discrepancy with the aim of using the decision boundary of a specific task to align the distribution of sources and targets. In general, a feature extractor *F* and two predictive classifiers *C*_1_ and *C*_2_ are included in the maximum mean discrepancy network architecture. In the training process, the discrepancy between classifiers *C*_1_ and *C*_2_ is maximized to detect the target samples close to the decision boundary. These samples are very easy to be mis predicted by the classifiers trained under source supervision. At the same time, feature extractor *F* is trained to generate a target representation that is far from the classification boundary and close to the source domain. This optimized process can be formulated as follows:

(4) $$\begin{array}{c} \left({\hat{\theta }}_{C_{1}},{\hat{\theta }}_{C_{2}}\right)=\arg\underset{\theta_{C_{1}},\;\theta_{C_{2}}}{\min}l\left({\hat{\theta }}_{F},\;\theta_{C_{1}},\theta_{C_{2}}\right) \end{array}$$

(5) $$\begin{array}{c} {\hat{\theta }}_{F}=\arg\underset{\theta_{F}}{\max}l\left(\theta_{F},\;{\hat{\theta }}_{C_{1}},{\hat{\theta }}_{C_{2}}\right) \end{array}$$

(6) $$\begin{array}{c} l\left(\theta_{F},\;\theta_{C_{1}},\theta_{C_{2}}\right)=\frac{1}{n_{s}}\overset{2}{\underset{j=1}{\sum }}\underset{x_{i}\in D^{s}}{\sum }J\left(C_{j}\left(F\left(x_{i}\right)\right),y_{i}\right)\\ -\frac{\lambda }{n_{t}}\underset{x_{i}\in D^{t}}{\sum }{\left|sf\left(C_{1}\left(F\left(x_{i}\right)\right)\right)sf\left(C_{2}\left(F\left(x_{i}\right)\right)\right)\right|}_{1} \end{array}$$

## 3. Proposed Method

### 3.1. Multi-Representation Domain Adaptation Network

**Figure 2:**
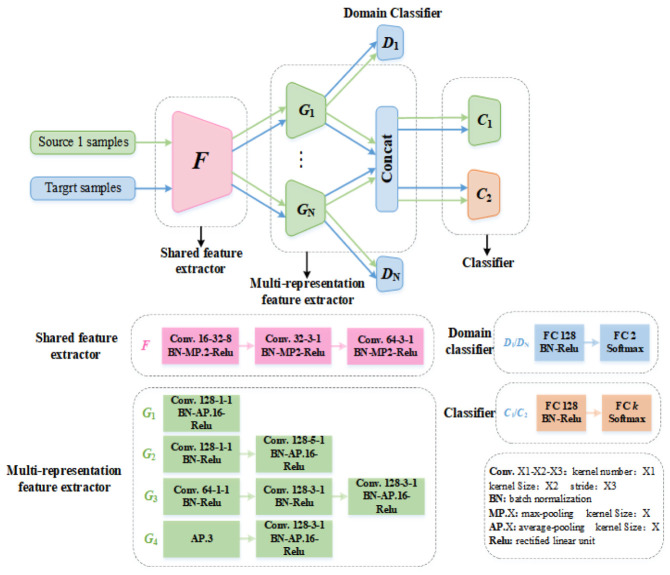
Integrated network architecture of the proposed method.

The integrated architecture of the proposed multi-representation network is shown in Figure 2. It mainly consists of four parts: the shared feature extractor, the multi-representation feature extractor, the domain classifier, and the state classifier. Specifically, the shared feature extractor includes three convolution layers. The first convolution layer uses a convolution core with a size of 32 to filter out interference noise, while the other two convolution layers use a convolution core with a size of 3 to extract common underlying characteristics. The multi-representation feature extractor module contains different network branches, each of which has a different network structure and convolution scale. It aims to extract the feature representation of specific characteristics from different angles. In this study, four different network structures are used as the multi-representation feature extractor, and the specific structure of each network branch *G_i_* is shown in Figure 2. A domain classifier is added after each representation network branch to judge the feature source of the network branch learning. Each characteristic obtained by the representation branch structure is spliced into a feature vector as the input of the state classifier, and the two classifiers are trained separately to distinguish different rolling mill running states. Meanwhile, the discrepancy between the two classifiers is used to detect the target samples close to the decision boundary. This process allows the feature extractor to learn a more robust characteristic representation during adversarial training.

### 3.2. Model Optimization

After the model is built, the specific optimization function should be designed to update the model parameters to achieve the expected diagnostic performance. Specifically, the loss objective function of the proposed model can be divided into three parts: supervised source domain classification loss, domain distinguishability loss in the multi-representation branch structure, and discrepancy loss of two classifiers. Under the supervised training of source domain samples, the two classifiers can independently learn to divide the decision boundary of fault classification. The loss function of the two classifiers can be expressed as follows:

(7) $$l_{c}=\frac{1}{n_{s}}\overset{2}{\underset{j=1}{\sum }}\underset{x_{i}\in D^{s}}{\sum }J\left(C_{j}\left(cat\left(G_{1}\left(F\left(x_{i}\right)\right),\ldots ,G_{4}\left(F\left(x_{i}\right)\right)\right)\right),y_{i}\right)$$

where cat represents vector connection operation.

In each multi-representation network branch, a domain classifier is used to perform adversarial training to realize the edge distribution matching represented by this feature; thus, the domain-invariant feature can be learned in this process. The domain adversarial loss of the multi-representation structure can be formulated as follows:

(8) $$\begin{array}{c} l_{d}=\frac{1}{n_{s}+n_{t}}\overset{4}{\underset{j=1}{\sum }}\underset{x_{i}\in D^{s}\cap D^{t}}{\sum }J\left(D_{j}\left(G_{j}\left(F\left(x_{i}\right)\right)\right),d_{i}\right) \end{array}$$

In addition to domain adversarial training, the second adversarial strategy of the proposed model is maximum classifier difference confrontation, which aims to use the predicted difference between the two classifiers to establish the relationship between the target sample and the task specific decision boundary. The two classifiers aim to detect the target samples far away from the source support, and the feature extractor is used to generate the target representation close to the source support. In this adversarial training process, more distinguished domain-invariant features can be learned. The maximum classifier discrepancy loss function of this model is given as follows:

(9) $$\begin{array}{c} l_{e}=\frac{1}{n_{t}}\sum_{x_{i}\epsilon D^{t}}\left|sf\left(C_{1}\left(cat\left(G_{1}\left(F\left(x_{i}\right)\right),\ldots ,G_{4}\left(F\left(x_{i}\right)\right)\right)\right)\right)\right.\\ {\left.-sf\left(C_{2}\left(cat\left(G_{1}\left(F\left(x_{i}\right)\right),\ldots ,G_{4}\left(F\left(x_{i}\right)\right)\right)\right)\right)\right|}_{1} \end{array}$$

By adding a GRL, the two adversarial training processes and source supervision training can be carried out synchronously, and the parameters of each module of the model can be updated synchronously. The total loss function of the proposed model is as follows:

(10) $$\begin{array}{c} l_{o}=l_{c}-\lambda \left(l_{d}+l_{e}\right) \end{array}$$

where the weight parameter *λ* changes gradually according to the formula $\lambda =\frac{2}{1+\exp\left(-\gamma \cdot p\right)}-1$ with *γ* being set to 10. In this study, p changes linearly from 0 to 1 with the training process.

According to the total loss function formula, the proposed parameter optimization problem of each module of the model can be expressed by the following formula:

(11) $$\begin{array}{c} {\hat{\theta }}_{F},{\hat{\theta }}_{G_{j}}{|}_{j=1}^{4}=\arg\left\{\;\min l_{c},\max l_{d},\min l_{e}\right\} \end{array}$$

(12) $$\begin{array}{c} {\hat{\theta }}_{D_{j}}{|}_{j=1}^{4}=\arg\left\{\;\min l_{d}\right\} \end{array}$$

(13) $$\begin{array}{c} {\hat{\theta }}_{C_{j}}{|}_{j=1}^{2}=\arg\left\{\;\min l_{c},\max l_{e}\right\} \end{array}$$

where ${\hat{\theta }}_{D_{j}},{\hat{\theta }}_{C_{j}}$ are the optimized values of $\theta_{D_{j}},\theta_{C_{j}}$, respectively.

Through the random gradient descent algorithm, the parameter update process for each network module is as follows:

(14) $$\begin{array}{c} \theta_{F}\leftarrow \theta_{F}-\eta \left(\frac{\partial l_{c}}{\partial \theta_{F}}-\frac{\partial l_{d}}{\partial \theta_{F}}+\frac{\partial l_{e}}{\partial \theta_{F}}\right) \end{array}$$

(15) $$\begin{array}{c} \theta_{G_{j}}\leftarrow \theta_{G_{j}}-\eta \left(\frac{\partial l_{c}}{\partial \theta_{G_{j}}}-\frac{\partial l_{d}}{\partial \theta_{G_{j}}}+\frac{\partial l_{e}}{\partial \theta_{G_{j}}}\right) \end{array}$$

(16) $$\begin{array}{c} \theta_{D_{j}}\leftarrow \theta_{D_{j}}-\eta \frac{\partial l_{d}}{\partial \theta_{D_{j}}} \end{array}$$

(17) $$\begin{array}{c} \theta_{C_{j}}\leftarrow \theta_{C_{j}}-\eta \left(\frac{\partial l_{c}}{\partial \theta_{C_{j}}}-\frac{\partial l_{e}}{\partial \theta_{C_{j}}}\right) \end{array}$$

where η is the learning rate, which is adjusted with the training progress according to the formula $\frac{\eta_{0}}{{\left(1+\alpha \cdot p\right)}^{\beta }}$, with *η*_0_ = 0.01, *α* = 10, and *β* = 0.75. This learning rate attenuation method helps the model rapidly converge to the optimal value [31].

**Figure 3:**
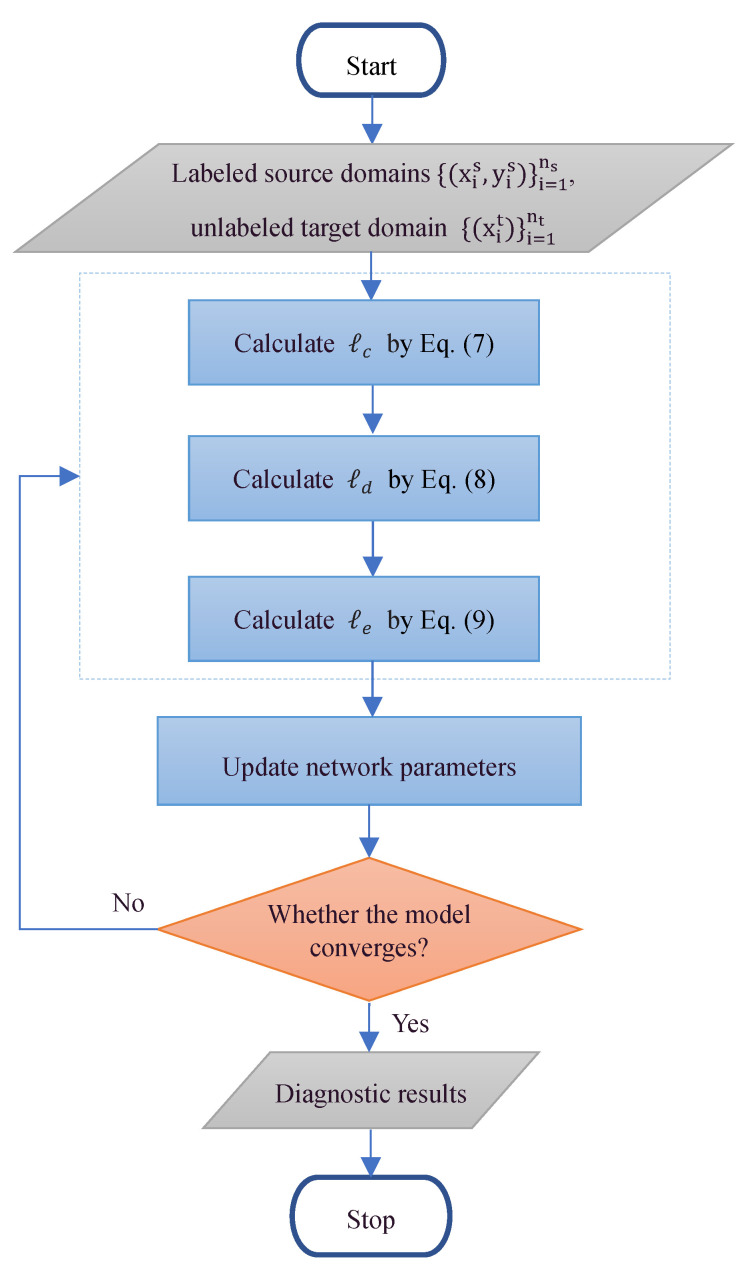
Training process of the proposed methods.

The overall training process of the proposed method is shown in Figure 3. The proposed method follows a simple end-to-end approach based on the standard unsupervised transfer learning training process. Only labeled source domain and unlabeled target domain samples are input into the network, and unlabeled target samples participate in the training. The total loss value in Equation (12) is obtained through forward calculation, and then the parameters in Equations (14)–(17) are optimized through the stochastic gradient descent (SGD) algorithm.

## 4. Experimental Study

In this section, by collecting the operating data of the bearing and reducer under different working conditions on a 4-H hot rolling mill test bench, an extensive experimental scheme was designed to verify the performance of the proposed method. The diagnostic results of several typical diagnostic models and the proposed method under the same experimental conditions are compared and analyzed.

### 4.1. Experimental Platform and Data Collection

#### 4.1.1. 4-H Hot Rolling Mill Experimental Platform

**Figure 4:**
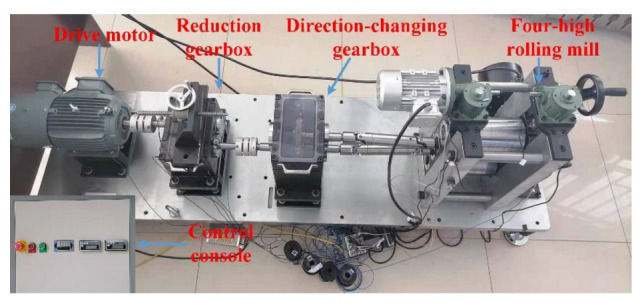
Four-high hot rolling mill experimental platform.

The overall structure of the 4-H hot rolling mill experimental platform is shown in Figure 4. It mainly includes a control console, a variable frequency adjustable speed drive motor, a reduction gearbox, a direction-changing gearbox, and a 4-H rolling mill. The control console is mainly composed of a variable-frequency motor controller, a loading motor controller, a pressure sensor display screen, and an emergency stop switch. The variable-frequency speed regulating motor is the driving source of the whole rolling mill system. The motor, reduction gearbox, and direction-changing gearbox are connected through couplings, and the direction-changing gearbox and 4-H rolling mill are connected through cross universal joints. The 4-H mill is composed of a mill stand, two backup rolls, and two working rolls. A loading device is installed at the top of the mill housing, which can exert pressure on the roll by electric or manual methods. Through the motor control button of the control console, the speed of the drive motor and the roll load can be adjusted to simulate different rolling conditions.

#### 4.1.2. Gearbox Dataset Description

**Figure 5:**
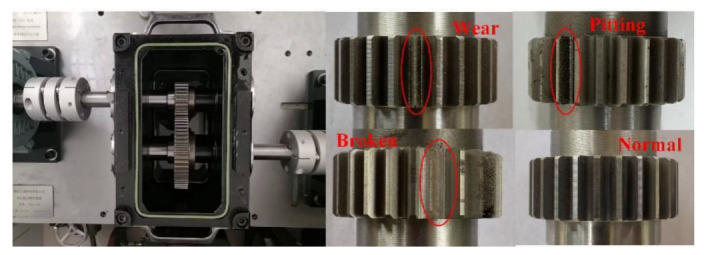
Reduction gearbox.

The gearbox data were collected on the reduction gearbox of the 4-H hot rolling mill experimental platform. As shown in Figure 5, the reduction gearbox includes two cylindrical spur gears—a large gear with 55 teeth and a small gear with 25 teeth. In the data acquisition experiment, the operating states of six health modes were simulated, including different single-point faults of large gears and small gears and composite faults of the two gears. The detailed health states are listed in Table 1. An acceleration sensor was placed on the reduction gearbox box to collect vibration signals. Gears with different failure modes were replaced in turn to simulate different gearbox operation states. The driving motor speed was controlled at 880× *g*, and three different load pressures were applied in turn to simulate different working conditions. The vibration signals collected under each load were used as a data source. The sampling frequency was set to 5120 Hz.

#### 4.1.3. Bearing Dataset Description

**Figure 6:**
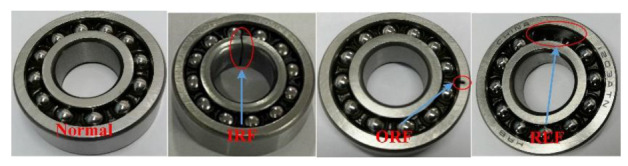
Bearings in different health states.

The bearing data were collected by monitoring the outer bearing of the working roll on the rolling mill, and the acceleration sensor was placed in the horizontal direction of the bearing seat. Four different bearing states were simulated: normal, inner ring fault (IRF), outer ring fault (ORF), and rolling element fault (REF). These faults were introduced in different parts of the rolling bearing through EDM, as shown in Figure 6. During data collection, the load pressure was constant, and the motor speed was set to 600, 840, and 1200× *g* to simulate different working conditions. The vibration signal collected at each speed was used as a data source. The sampling frequency of the acquisition card was set to 10,240 Hz.

### 4.2. Experimental Setup

For the data of each failure mode under different working conditions, we used a sliding window with a size of 1024 to intercept samples. In the gearbox dataset, 300 samples were obtained for each fault class; there were 1800 samples under each working condition. In the bearing dataset, 200 samples were obtained for each fault class, including a total of 800 samples under each working condition. In each diagnostic task, 50% of the samples were randomly selected as the training set and the remaining 50% as the test set. Because the data under each working condition was used as a source, in the experiment, a data source was randomly selected as the source domain in cross-domain fault diagnosis, and the remaining data sources were selected in turn as the target domain to be diagnosed. In this study, a total of 12 diagnostic tasks were set, and the detailed information is shown in Table 2. In the process of model training, the size of the mini batch was set to 32, and a total of 20 epochs were trained. In addition, several typical diagnostic methods were introduced to compare the performance of the proposed model with its actual performance. They are briefly described as follows:

(1) Convolutional neural network (CNN): CNN implements the traditional supervised learning paradigm without adding a domain-adaptive algorithm. It uses the source domain for training and directly tests in the target domain.
(2) Deep adaptation network (DAN) [32]: DAN is a domain-adaptive depth network that uses the MMD to minimize the difference in edge distribution.
(3) Deep CORAL (D-CORAL) [33]: D-CORAL is a deep domain-adaptive network that uses the coral algorithm to align the second-order statistical characteristics of the source and target domains.
(4) Domain adversarial neural network (DANN) [29]: DANN is a deep domain-adaptive network that generates domain-invariant characteristics by applying adversarial training.
(5) Joint adaptation network (JAN) [34]: JAN is a domain-adaptive depth network that uses joint MMD (JMMD) to minimize joint distribution discrepancy.
(6) Multi-adversarial domain adaptation (MADA) [35]: MADA is a deep domain-adaptive network that applies a multi-pair adversarial domain adaptation algorithm to learn domain-invariant representation.

For a fair comparison, all comparison methods used the same network parameters as the proposed model. To avoid the effect of random factors, each trial was repeated 10 times, and the average diagnostic results were adopted.

### 4.3. Result Discussion and Analysis

#### 4.3.1. Diagnosis Result Discussion

**Figure 7:**
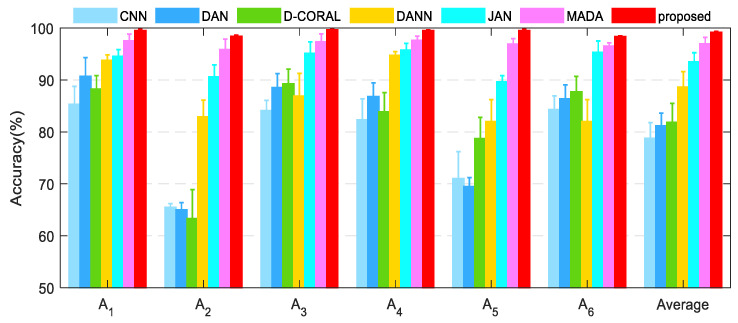
Diagnosis results of the different methods on the gearbox dataset.

In this section, the diagnostic results of the proposed method and other comparative methods on different diagnostic tasks are presented and discussed. The diagnostic results of different methods from the gearbox dataset are shown in Figure 7, and specific diagnostic accuracy and standard deviation are listed in Table 3. Clearly, the diagnostic performance of the proposed method on six diagnostic tasks of the gearbox dataset was better than that of other comparative methods. Because a domain-adaptive algorithm is not applied, the CNN achieved the lowest average diagnostic accuracy of 78.76% on the six diagnostic tasks. As the distributed difference measurement algorithm is introduced in the DAN and D-CORAL, their diagnostic performance slightly improves compared with the CNN, with their average diagnostic accuracy reaching 81.15% and 81.85%, respectively. However, on diagnostic task A_2_, the DAN and D-CORAL showed a negative transfer phenomenon, and their diagnostic accuracy was lower than that of the CNN. The DANN still achieved 82.88% diagnostic accuracy, indicating that the DANN with a domain adversarial strategy can better mitigate the effect of negative transfer than the DAN and D-CORAL. The JAN and MADA consider the conditional distribution domain adaptation; therefore, their diagnostic performance is significantly improved compared with that of the global distribution domain-adaptive method. Their average diagnostic accuracy in the six diagnostic tasks reached 93.50% and 96.96%, respectively; however, when using a single-feature representation for domain adaptation, the JAN and MADA may lose some important diagnostic information, and the diagnostic performance degrades. The proposed method migrates information from the perspective of multi-feature representation and considers the decision boundary division of the target task. The joint distribution difference between the source domain and the target domain can be well compensated. Therefore, the proposed method obtained the highest average diagnostic accuracy in this diagnostic task, which is 2.19% higher than the most competitive MADA, and showed the best model stability.

**Figure 8:**
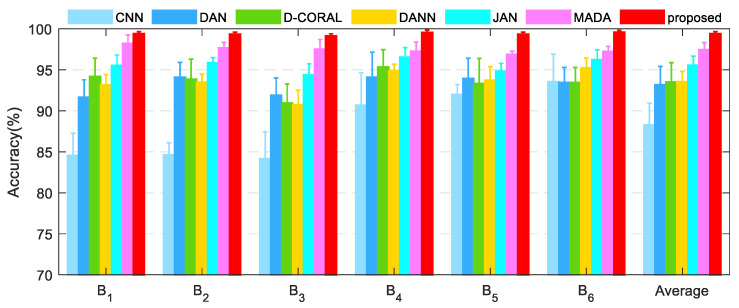
Diagnosis results of the different methods on the bearing dataset.

The diagnostic results of different methods on the diagnostic task of the rolling mill bearing dataset are shown in Figure 8 and Table 4. Similar to the aforementioned case, the proposed method achieved the best diagnostic performance on the bearing dataset, and the average diagnostic accuracy on the six diagnostic tasks of the bearing dataset was 99.40%. The DAN, D-CORAL, and DANN obtained similar diagnostic accuracy, which were 4.92%, 5.26%, and 5.27% higher than that of the CNN, respectively. Compared with the DAN, D-CORAL, and DANN, the JAN and MADA showed better diagnostic performance improvement, with their average diagnostic accuracy reaching 95.57% and 97.47%, respectively. The shift from global distribution matching to conditional distribution matching is the key to improving diagnostic performance. The proposed method further considers the multi-representation diagnostic information transfer and the target decision boundary division, which further improves the diagnostic accuracy and reliability of cross-domain diagnostic tasks.

#### 4.3.2. Visualization Results

To compare the diagnostic performance of the proposed method with that of several typical methods more clearly, this section presents several visualization results on diagnostic tasks A_3_ and B_3_.

**Figure 9:**
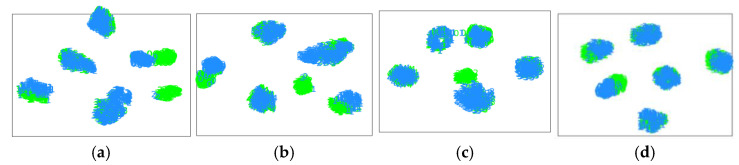
Feature visualization of the different methods on the gearbox dataset. (**a**) CNN-taskA_3_ (**b**) DAN-taskA_3_ (**c**) DANN-taskA_3_ (**d**) Proposed-taskA_3_.

**Figure 10:**
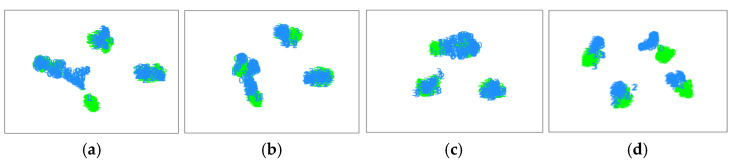
Feature visualization of the different methods on the bearing dataset. (**a**) CNN-taskB_3_ (**b**) DAN-taskB_3_ (**c**) DANN-taskB_3_ (**d**) Proposed-taskB_3_.

First, the t-distributed stochastic neighbor embedding (t-SNE) algorithm [36] was applied to intuitively understand the transfer learning process of diagnostic knowledge. The high-level representation learned by the feature extractor is plotted directly after dimensionality reduction. The values in green denote source instances, and those in blue denote target instances. Figure 9 shows the feature distribution of the proposed method and the CNN, DAN, and DANN on the gearbox dataset for diagnosis task A_3_. It can be seen that category-level distribution differences of varying degrees exist in the high-level characteristics of the CNN, DAN, and DANN. Specifically, the characteristic distributions of category 2 samples in the source and target domains are not well matched. This is because the same fault mode exists for large gear tooth breakage and small gear wear, which easily causes feature confusion. Multi-representation feature learning and duplex adversarial strategies are used to extract features from multiple perspectives and clarify the clear division of the target decision boundary, as shown in Figure 9d. The proposed method can compensate for the lack of diagnostic knowledge, match the feature distribution of each fault state, and accurately transfer diagnostic knowledge. Similarly, on diagnostic task B_3_, the proposed method still achieved the best migration effect. As shown in Figure 10, the CNN, DAN, and DANN had serious feature distribution aliasing, which will greatly reduce the diagnostic accuracy.

**Figure 11:**
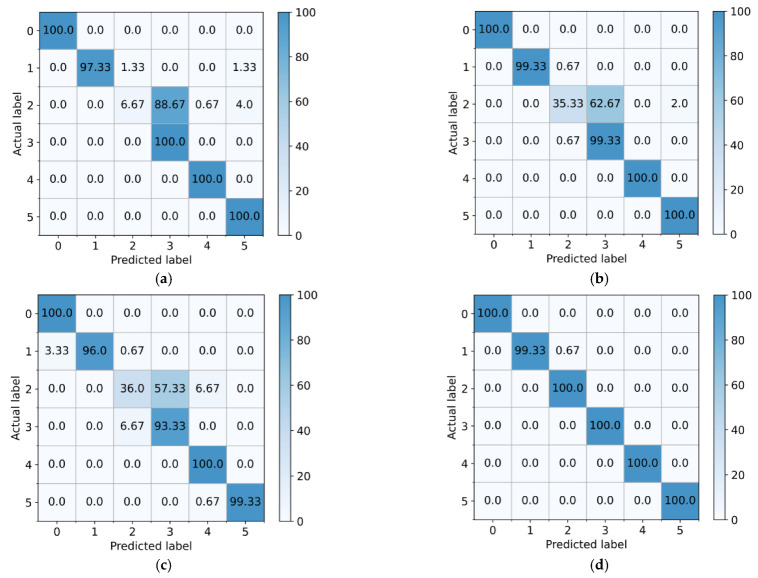
Confusion matrix of the different methods on the gearbox dataset (%) (**a**) CNN-taskA_3_ (**b**) DAN-taskA_3_ (**c**) DANN-taskA_3_ (**d**) Proposed-taskA_3_.

**Figure 12:**
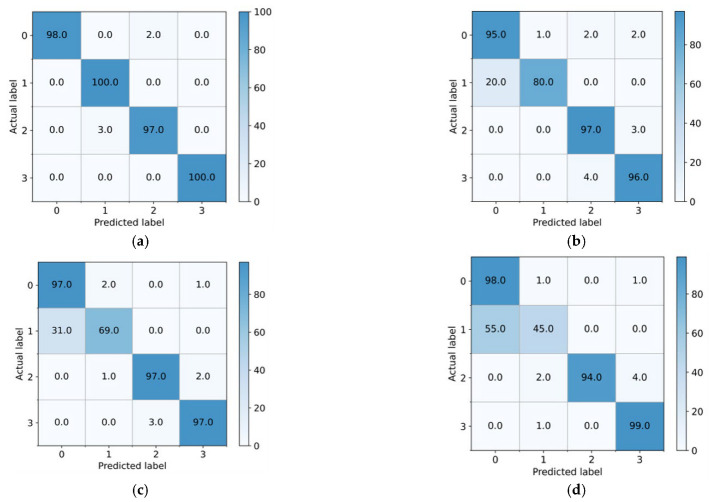
Confusion matrix of the different methods on the bearing dataset (%) (**a**) CNN-taskB_3_ (**b**) DAN-taskB_3_ (**c**) DANN-taskB_3_ (**d**) Proposed-taskB_3_.

According to the visual characteristics of learning, the confusion matrixes of the proposed method and the three comparison methods are further displayed. As shown in Figure 11, due to the category-level distribution deviation of transfer characteristics, almost all samples with the large gear tooth breakage fault of the CNN are incorrectly divided into large gear tooth breakage and small gear wear fault modes. Although the misclassification of the DAN and DANN in the fault category of broken large gear teeth was alleviated, the diagnosis rates were still too low at only 35.33% and 36%, respectively. The proposed method not only achieves a 100% diagnosis rate for the large gear tooth broken fault mode but also obtains satisfactory recognition accuracy for other fault categories. In diagnosis task B_3_, as shown in Figure 12, which corresponds to the characteristic distribution shown in Figure 10, the CNN, DAN, and DANN misdiagnosed the inner ring fault as normal to varying degrees, causing the machine to run with the fault; hence, these methods have poor fault diagnosis. The proposed method can correctly identify the fault mode in the inner ring fault and does not divide any fault samples into normal states, proving the reliability of the proposed method’s diagnosis performance.

**Figure 13:**
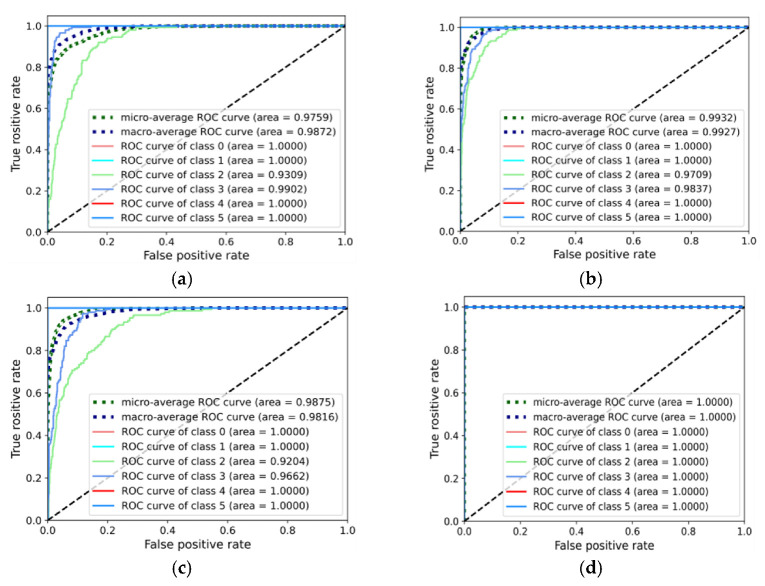
ROC curves of the different methods on the gearbox dataset (**a**) CNN-taskA_3_ (**b**) DAN-taskA_3_ (**c**) DANN-taskA_3_ (**d**) Proposed-taskA_3_.

**Figure 14:**
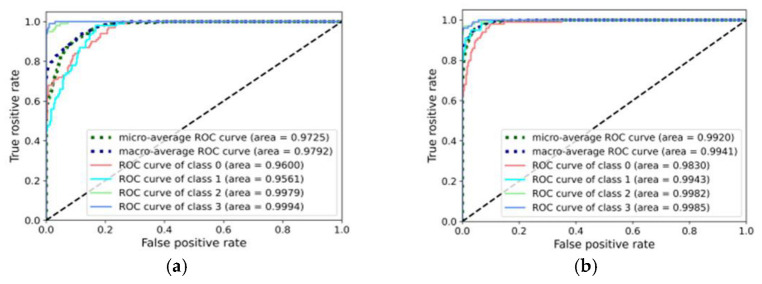

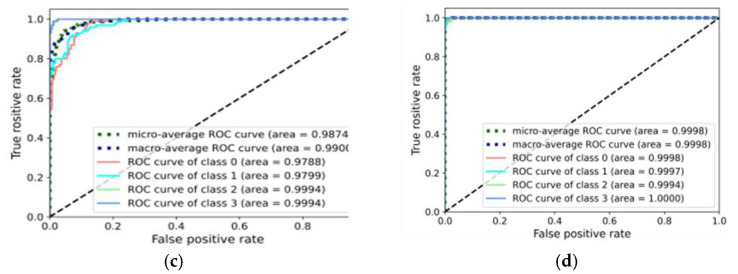
ROC curves of the different methods on the bearing dataset (**a**) CNN-taskB_3_ (**b**) DAN-taskB_3_ (**c**) DANN-taskB_3_ (**d**) Proposed-taskB_3_.

Finally, the model sensitivity and stability of different methods are analyzed, as shown in Figure 13 and Figure 14. These figures show the receiver operating characteristic (ROC) curves of diagnostic tasks A_3_ and B_3_ with the proposed methods, CNN, DAN, and DANN. Clearly, the area under the curve (AUC) of the proposed method for each fault category was basically close to 1, and the CNN obtained the minimum AUC, followed by the DAN and DANN. This confirms that the introduction of the domain-adaptive algorithm can improve the diagnosis performance to a certain extent in the fault diagnosis task under variable conditions; however, only considering the edge distribution matching is not enough. The multi-representation feature extraction mechanism and dual adversarial strategy of the proposed method realize more comprehensive learning of diagnostic knowledge and accurate transfer, and thus the proposed method has high sensitivity and stability.

## 5. Conclusions

This study developed a multi-representation domain adaptation network with duplex adversarial learning for rolling mill fault diagnosis under varying working conditions. The proposed method can extract comprehensive features and perform accurate knowledge transfer to realize high-performance fault diagnosis of key components of the hot rolling mill. Specifically, a multi-representation network structure was designed to extract rolling mill equipment status information from multiple perspectives. Then, the domain adversarial strategy was adopted to match the source and target domains of each pair of representations for learning the domain-invariant features from multiple representations. In addition, maximum classifier diversity was adopted to generate target features that are close to the source support, thus forming a robust decision boundary. Extensive experiments were carried out on the reducer and roll bearing fault state data set of a four-high rolling mill experimental platform. The average diagnostic rates of the proposed method on different diagnostic tasks reached 99.15% and 99.40%, which were 2.19% and 1.93% higher than the rates of the most competitive method, respectively. Furthermore, t-SNE feature visualization, the confusion matrix, and the ROC curve were applied to intuitively display the implementation results of the proposed method. The experimental results showed that the proposed duplex adaptive multi-representation domain adaptation method can effectively diagnose knowledge transfer from multiple perspectives and divide clear fault category decision boundaries. The proposed method is superior to other domain-adaptive methods in model stability and fault identification accuracy and can realize effective fault diagnosis of rolling mill equipment under variable working conditions.

Although the experiments for this method showed good diagnostic accuracy achieved by the proposed method, only four different representation subnets were used for multi-view feature extraction. To gain a better understanding of feature representation from more perspectives, more network branches at different scales are needed. However, that will inevitably increase the computational complexity of the network as well as require more sample training to avoid overfitting. Therefore, further research is necessary to learn representation features from more perspectives and to design lightweight networks.

## Figures and Tables

**Table 1:** Description of the health conditions of the reduction gearbox.

Label	Condition
0	Normal
1	Large gear pitting
2	Large gear tooth breakage
3	Large gear tooth breakage and small gear wear
4	Large gear pitting and small gear wear
5	Small gear wear

**Table 2:** Description of the cross-domain diagnosis tasks.

Dataset	Task	Source	Target	Dataset	Task	Source	Target
Gearbox	A1	load0	load1	Bearing	B1	600× *g*	840× *g*
	A2	load0	load2		B2	600× *g*	1200× *g*
	A3	load1	load0		B3	840× *g*	600× *g*
	A4	load1	load2		B4	840× *g*	1200× *g*
	A5	load2	load0		B5	1200× *g*	600× *g*
	A6	load2	load1		B6	1200× *g*	840× *g*

**Table 3:** Average diagnostic accuracy (%) and standard deviation of different methods on the gearbox dataset.

Task	CNN	DAN	D-CORAL	DANN	JAN	MADA	Proposed
A1	85.30 ± 3.47	90.70 ± 3.63	88.24 ± 2.61	93.80 ± 1.05	94.53 ± 1.30	97.52 ± 1.33	99.51 ± 0.25
A2	65.48 ± 0.71	65.01 ± 1.36	63.30 ± 5.57	82.88 ± 3.25	90.62 ± 2.28	95.86 ± 2.01	98.38 ± 0.26
A3	84.10 ± 1.98	88.53 ± 2.71	89.26 ± 2.83	86.91 ± 4.35	95.13 ± 2.21	97.36 ± 1.54	99.64 ± 0.23
A4	82.33 ± 4.02	86.80 ± 2.64	83.85 ± 3.70	94.75 ± 0.70	95.75 ± 1.32	97.62 ± 0.85	99.51 ± 0.16
A5	71.02 ± 5.19	69.48 ± 1.70	78.72 ± 4.07	81.98 ± 4.24	89.62 ± 1.22	96.89 ± 1.06	99.49 ± 0.31
A6	84.30 ± 2.66	86.37 ± 2.68	87.72 ± 2.97	81.98 ± 4.24	95.32 ± 2.19	96.53 ± 0.61	98.35 ± 0.14
Average	**78.76 ± 3.01**	**81.15 ± 2.45**	**81.85 ± 3.63**	**88.62 ± 2.99**	**93.50 ± 1.75**	**96.96 ± 1.23**	**99.15 ± 0.23**

**Table 4:** Average diagnostic accuracy (%) and standard deviation of different methods on the bearing dataset.

Task	CNN	DAN	D-CORAL	DANN	JAN	MADA	Proposed
B1	84.54 ± 2.72	91.65 ± 2.13	94.20 ± 2.22	93.15 ± 1.25	95.54 ± 1.26	98.21 ± 1.05	99.40 ± 0.25
B2	84.65 ± 1.45	94.10 ± 1.81	93.85 ± 2.44	93.50 ± 0.97	95.88 ± 0.61	97.68 ± 0.65	99.35 ± 0.25
B3	84.15 ± 3.31	91.90 ± 2.11	90.95 ± 2.32	90.75 ± 1.76	94.37 ± 1.36	97.53 ± 1.16	99.15 ± 0.24
B4	90.70 ± 3.93	94.10 ± 3.05	95.35 ± 2.10	94.86 ± 0.83	96.54 ± 1.15	97.26 ± 1.12	99.55 ± 0.29
B5	92.00 ± 1.20	93.95 ± 2.47	93.35 ± 3.04	93.75 ± 1.64	94.83 ± 0.96	96.88 ± 0.38	99.35 ± 0.25
B6	93.55 ± 3.34	93.45 ± 1.83	93.45 ± 1.83	95.22 ± 1.19	96.24 ± 1.18	97.25 ± 0.59	99.60 ± 0.20
Average	**88.27 ± 2.66**	**93.19 ± 2.23**	**93.53 ± 2.33**	**93.54 ± 1.27**	**95.57 ± 1.09**	**97.47 ± 0.83**	**99.40 ± 0.25**

## Data Availability

Not applicable.

## Notations List

**Notations**	**Instructions**
$D^{s}$	source domain
$n_{s}$	number of source domain samples
$y_{i}^{s}$	health state label
$D^{t}$	target domain
$n_{t}$	number of target domain samples
$P\left(X^{s}\right)$	source domain distribution
$P\left(X^{t}\right)$	target domain distribution
*F*	feature extractor
*D*	domain classifier
*C*	Classifier
*θ_F_*	feature extractor parameters
*θ_D_*	domain classifier parameters
*θ_C_*	classifier parameters
${\hat{\theta }}_{F}$	optimized values of *θ_F_*
${\hat{\theta }}_{D}$	optimized values of *θ_D_*
${\hat{\theta }}_{C}$	optimized values of *θ_C_*
λ	trade-off parameter
η	learning rate

## References

[1] Yin S., Ding S., Xie X., Luo H. (2014). A review on basic data-driven approaches for industrial process monitoring. IEEE Trans. Ind. Electron..

[2] Zhang Y., Mu L., Shen G., Yu Y., Han C. (2018). Fault diagnosis strategy of CNC machine tools based on cascading failure. J. Intell. Manuf..

[3] Feng Z., Chen X., Wang T. (2017). Time-varying demodulation analysis for rolling bearing fault diagnosis under variable speed conditions. J. Sound Vib..

[4] Luo H., Li K., Kaynak O., Yin S., Huo M., Zhao H. (2020). A robust data-driven fault detection approach for rolling mills with unknown roll eccentricity. IEEE Trans. Control Syst. Technol..

[5] Peng R., Zhang X., Shi P. (2022). Bearing fault diagnosis of hot-rolling mill utilizing intelligent optimized self-adaptive deep belief network with limited samples. Sensors.

[6] Lei Y., Yang B., Jiang X., Jia F., Li N., Nandi A. (2020). Applications of machine learning to machine fault diagnosis: A review and roadmap. Mech. Syst. Signal Process..

[7] Liu R., Yang B., Zio E., Chen X. (2018). Artificial intelligence for fault diagnosis of rotating machinery: A review. Mech. Syst. Signal Process..

[8] Jia F., Lei Y., Lin J., Zhou X., Lu N. (2016). Deep neural networks: A promising tool for fault characteristic mining and intelligent diagnosis of rotating machinery with massive data. Mech. Syst. Signal Process..

[9] Zhang Y., Li X., Gao L., Chen W., Li P. (2019). Intelligent fault diagnosis of rotating machinery using a new ensemble deep auto-encoder method. Measurement.

[10] Shao S., Yan R., Lu Y., Wang P., Robert X. (2020). DCNN-based multi-signal induction motor fault diagnosis. IEEE Trans. Instrum. Meas..

[11] Han D., Tian J., Xue P., Shi P. (2021). A novel intelligent fault diagnosis method based on dual convolutional neural network with multi-level information fusion. J. Mech. Sci. Technol..

[12] Jia F., Lei Y., Guo L., Lin J., Xing S. (2018). A neural network constructed by deep learning technique and its application to intelligent fault diagnosis of machines. Neurocomputing.

[13] Shi P., Yu Y., Gao H., Hua C. (2022). A novel multi-source sensing data fusion driven method for detecting rolling mill health states under imbalanced and limited datasets. Mech. Syst. Signal Process..

[14] Yang D., Karimi H.R., Sun K. (2021). Residual wide-kernel deep convolutional auto-encoder for intelligent rotating machinery fault diagnosis with limited samples. Neural Netw..

[15] Yu Y., Shi P., Tian J., Xu X., Hua C. (2022). Rolling mill health states diagnosing method based on multi-sensor information fusion and improved DBNs under limited datasets. ISA Trans..

[16] Li W., Huang R., Li J., Liao Y., Chen Z., He G., Yan R., Gryllias K. (2022). A perspective survey on deep transfer learning for fault diagnosis in industrial scenarios: Theories, applications and challenges. Mech. Syst. Signal Process..

[17] Li C., Zhang S., Qin Y., Estupinan E. (2020). A systematic review of deep transfer learning for machinery fault diagnosis. Neurocomputing.

[18] Tian J., Han D., Li M., Shi P. (2022). A multi-source information transfer learning method with subdomain adaptation for cross-domain fault diagnosis. Knowl. Based. Syst..

[19] Pan S., Yang Q. (2010). A survey on transfer learning. IEEE Trans. Knowl. Data Eng..

[20] Jiao J., Zhao M., Lin J., Liang K. (2020). Residual joint adaptation adversarial network for intelligent transfer fault diagnosis. Mech. Syst. Signal Process..

[21] Li X., Zhang W., Ding Q., Sun J. (2019). Multi-Layer domain adaptation method for rolling bearing fault diagnosis. Signal Process.

[22] Qian Q., Qin Y., Wang Y., Liu F. (2021). A new deep transfer learning network based on convolutional auto-encoder for mechanical fault diagnosis. Measurement.

[23] Li X., Zhang W., Xu N., Ding Q. (2020). Deep learning-based machinery fault diagnostics with domain adaptation across sensors at different places. IEEE Trans. Ind. Electron..

[24] Han T., Liu C., Yang W., Jiang D. (2018). Deep transfer network with joint distribution adaptation: A new intelligent fault diagnosis framework for industry application. ISA Trans..

[25] Li F., Tang T., Tang B., He Q. (2020). Deep convolution domain-adversarial transfer learning for fault diagnosis of rolling bearings. Measurement.

[26] Guo L., Lei Y., Xing S., .Yan T., Li N. (2019). Deep convolutional transfer learning network: A new method for intelligent fault diagnosis of machines with unlabeled data. IEEE Trans. Ind. Electron..

[27] Lu W., Liang B., Meng D., Tao Z. (2016). Deep model based domain adaptation for fault diagnosis. IEEE Trans. Ind. Electron..

[28] Zhu Y., Zhuang F., Wang J., Chen J., Shi Z., Wu W., He Q. (2019). Multi-representation adaptation network for cross-domain image classification. Neural Netw..

[29] Yaroslov G., Evgeniya U., Hana A., Pascal G., Hugo L., Francois L., Mario M., Victor L. (2016). Domain-adversarial training of neural networks. J. Mach. Learn. Res..

[30] Saito K., Watanabe K., Ushiku Y., Harada T. Maximum classifier discrepancy for unsupervised domain adaptation. Proceedings of the 2018 IEEE/CVF Conference on Computer Vision and Pattern Recognition.

[31] Loshchilov I., Hutter F. (2016). Sgdr: Stochastic gradient descent with warm restarts. arXiv.

[32] Long M., Cao Y., Wang J., Jordan M.I. Learning transferable features with deep adaptation networks. Proceedings of the 32nd International Conference on International Conference on Machine Learning (JMLR).

[33] Sun B., Saenko K. Deep CORAL: Correlation alignment for deep domain adaptation. Proceedings of the14th European Conference on Computer Vision (ECCV).

[34] Long M., Wang J., Wang J., Jordan M.I. Deep transfer learning with joint adaptation networks. Proceedings of the 34th International Conference on Machine Learning.

[35] Pei Z., Cao Z., Long M., Wang J. Multi-adversarial domain adaptation. Proceedings of the 32nd AAAI Conference on Artificial Intelligence/30th Innovative Applications of Artificial Intelligence Conference/8th AAAI Symposium on Educational Advances in Artificial Intelligence.

[36] Van der Maaten L., Hinton G. (2008). Visualizing data using t-SNE. J. Mach. Learn. Res..

